# Association between prenatal exposure to antihypertensive medication and neurodevelopmental and educational outcomes in children

**DOI:** 10.1038/s41598-025-22887-2

**Published:** 2025-11-06

**Authors:** Shrifah Alkhalaf, Sarjit Singh, Jill P. Pell, Scott M. Nelson, Daniel F. Mackay, Michael Fleming

**Affiliations:** 1 School of Health and Wellbeing, University of Glasgow, Glasgow, UK; 2 School of Medicine, Dentistry and Nursing, University of Glasgow, Glasgow, UK; 3 School of Health and Wellbeing, College of Medical, Veterinary and Life Sciences, University of Glasgow, Clarice Pears Building, 90 Byres Road, Glasgow, G12 8TB UK

**Keywords:** Antihypertensive agents, Pregnancy, Child development, Education, Hypertension disorders., Neurodevelopmental disorders, Epidemiology, Drug safety, Clinical pharmacology

## Abstract

Hypertensive disorders of pregnancy (HDP) require medication but neurodevelopmental consequences of prenatal exposure to antihypertensive medication are unclear. We linked five national health and education databases to conduct a cohort study of 179,024 children born in Wales between 2009 and 2016 and investigated associations between maternal HDP and antihypertensive medication and educational and neurodevelopmental outcomes, adjusting for sociodemographic, maternal, and pregnancy confounders. Among, 3,440 (1.9%) mothers who had HDP or who received antihypertensive medication during pregnancy; 629 (0.3%) had treated HDP, 950 (0.5%) untreated HDP, and 1861 (1.0%) received antihypertensive medication without documented HDP. Autistic spectrum disorder (ASD) was associated with prenatal exposure to antihypertensive medication (adjusted OR 1.67, 95% CI 1.26–2.21, *p* < 0.001); specifically, beta-blockers (adjusted OR 1.96, 95% CI 1.39–2.78). Untreated maternal HDP was not associated with ASD. Learning and communication difficulties were associated with beta-blockers and methyldopa, as well as untreated HDP, suggesting confounding by indication. Neither HDP nor anti-hypertensive medication were associated with attention deficit hyperactivity disorder (ADHD). In this large, unselected cohort, prenatal beta-blocker exposure was associated with ASD independent of maternal hypertension. Further research should be undertaken regarding the biological mechanisms and choice of antihypertensive therapy in pregnancy should be carefully selected to balance maternal benefits and potential neurodevelopmental risks in offspring.

**Supplementary Information:**

The online version contains supplementary material available at 10.1038/s41598-025-22887-2.

## Introduction

Hypertension disorders of pregnancy (HDP), encompassing chronic hypertension, gestational hypertension, and pre-eclampsia, affect up to 10% of pregnancies worldwide^1^. Offspring of mothers with HDP are at increased risk of intra-uterine growth restriction and preterm delivery^1,2^, and adverse neurodevelopmental conditions including autistic spectrum disorder (ASD), attention deficit hyperactivity disorder (ADHD), cerebral palsy, and intellectual disabilities^3,4^. In pre-eclampsia specifically, these outcomes may be driven by impaired cerebral angiogenesis and neurogenesis in the developing fetus^5^. 

Chronic hypertension is a major risk factor for pre-eclampsia and, if untreated, can lead to life-threatening complications such as eclampsia, Hemolysis, Elevated Liver Enzymes, Low Platelet Count (HELLP) syndrome, and placental abruption^6,7^. Globally, pre-eclampsia contributes to over 500,00 fetal and neonatal deaths and 70,000 maternal deaths annually,^7^ highlighting the necessity of antihypertensive treatment during pregnancy. However, emerging evidence suggests that in-utero exposure to antihypertensive medication itself may influence neurodevelopmental trajectories in offspring^8^.

Concerns have been raised regarding specific antihypertensive agents. A randomised clinical trial of 170 children reported poorer fine motor function following prenatal exposure to nifedipine^9^. Methyldopa exposure has been associated with lower Intelligence Quotient (IQ) scores in a Canadian, prospective cohort study,^10^ and to sleeping disorders and poorer gross motor outcomes in a retrospective study of 202 Dutch children^11^. Observational studies have associated ADHD with prenatal beta-blocker exposure,^11^ and ASD with prenatal exposure to beta-blockers and calcium channel blockers^12^.

A recent large-scale Tohoku Medical Megabank Project of 5934 mother-child pairs did not investigate antihypertensive treatment but demonstrated that children born to mothers with HDP had 29% increased risk of developmental delays at 24 months of age^13^. The association was mediated by factors including preterm birth, intensive care unit admission, and head circumstance suggesting that several direct and indirect pathways contribute to any observed association.

A review by Koren et al.^14^ concluded no adverse effect of labetalol and methyldopa use in pregnancy on long-term neurodevelopmental outcomes in offspring, however previous studies have not investigated maternal hypertension and antihypertensive therapy simultaneously. Conversely, a meta-analysis study observed a significant association between HDP and ASD and ADHD, without considering adjustment for medication^15^. Therefore, confounding may persist, influencing any previously observed associations between HDP or antihypertensive therapy and neurodevelopmental outcomes. Additionally, previous studies have been small-scale, heterogeneous in medication classes and outcomes, and inconsistent in adjusting for confounders, leading to conflicting results^14^ and making it difficult to distinguish whether observed effects stem from medication effects or the underlying HDP^15^.

This study aimed to bridge previous gaps by investigating the associations between treated and untreated HDP during pregnancy and a comprehensive range of neurodevelopmental and educational outcomes in a large, unselected population cohort.

## Methods

### Study design and data sources

A population-based retrospective cohort study was conducted using routinely collected data on schoolchildren born in Wales between 2009 and 2016. The Secure Anonymised Information Linkage (SAIL) databank provided linked, pseudonymised data extracted from five health and education databases: the Annual District Birth Extract (ADBE, containing birth registration data for children born in Wales); the National Community Child Health Databases (NCCHD, containing maternal, birth and child health surveillance data for children resident in Wales); the Welsh Longitudinal General Practice Dataset (WLGP, containing national-level primary care encounters and prescriptions); the Maternity Indicator Dataset (MIDS, containing additional obstetric and delivery information); and the Education Dataset Wales (EDUW, containing school census data, attendance, attainment and special educational needs). School and pupil data for Wales covers state funded learning centres and contains several datasets including information from the Pupil Level Annual School Census (PLASC) and the Welsh Examinations Database (WED) (see Supplementary File S1 online).

### Inclusion and exclusion criteria

The cohort comprised all live births in Wales between 2009 and 2016, where data were available for both mother and child and where the child attended school in Wales between 2014 and 2021 and had valid linked education data.

To ensure comprehensive capture of medical history, included mothers were required to have continuous registration with a SAIL-participating practice for at least two years prior to delivery. SAIL primary care data were available from 2007; therefore, deliveries were analysed from 2009 to ensure a 2-year lookback pre-delivery. Education data, including SEN outcomes, were complete until 2021; therefore, we included children born up to 2016 as most children born after this date would not be in school by 2021. As a result, children were followed up from birth for a maximum of 13 years. Singleton, twin, and higher order multiple births were included, as well as serial births from the same mother during the study period. We excluded pregnancies resulting in miscarriage, abortion, or stillbirth and extreme outlier values were treated as missing data: birthweight < 100 g or > 5,500 g; gestational age at delivery < 24 or ≥ 44 weeks; and maternal age < 11 years.

### Exposures

HDP were identified using relevant Read Clinical Classification System (READ) codes (Supplementary Table S1 online) recorded within the two years prior to delivery. Prenatal anti-hypertensive medication exposure was defined as receipt of at least one prescription for an antihypertensive agent from one month before conception to the date of delivery (Supplementary Table S2 online). Date of conception was derived by subtracting gestational age at delivery plus two weeks from date of delivery.

We included mothers of any age with HDP or who received antihypertensive medication during pregnancy. Gestational hypertension is typically diagnosed if mothers’ blood pressure >= 140/90 mmHg after 20 weeks of gestation with no proteinuria, while pre-eclampsia or superimposed pre-eclampsia occurs with proteinuria >= 2 + in the dipstick test. Pre-existing chronic hypertension is diagnosed before pregnancy or before 20 weeks of gestion^13^. We did not adjust for the co-morbidities and polypharmacy.

### Outcomes

The outcomes were recorded at school and therefore when children were aged between 5 and 13 years. The primary outcome of interest was special educational need (SEN) recorded in the EDUW database and defined as the requirement for additional educational support beyond that expected for age-matched peers. Children with SEN have significant learning difficulties or disabilities which prevent them from using educational facilities independently, necessitating specialised educational provision. There is no specific diagnostic age; identification can occur from birth through to young adulthood aged up to 25. Early identification improves long-term outcomes for the child^16^. Secondary outcomes included specific SEN categories recorded within EDUW: ASD, sensory impairment (visual, hearing or multi-sensory impairments), communication problems (speech or language difficulties), learning difficulties, physical and medical difficulties, and social, emotional and behavioural difficulties. SEN status was assessed annually, and children could have multiple SEN categories recorded. In addition, we examined ADHD, ascertained either through SEN records at school or a WLGP documented diagnosis or relevant medication prescription at any time in the study period following birth (Supplementary Table S3 online). Since part of this analysis relied on primary care data, children were excluded from the ADHD analyses if they did not have complete primary care record coverage from age 5 years until the end of follow-up.

### Covariates

Potential confounders obtained from the NCCHD databases included sociodemographic factors (child’s sex, ethnicity, age and socioeconomic deprivation), maternal factors (maternal age and maternal smoking status, and pregnancy characteristics (parity and multiple births). Potential mediators included mode of delivery, gestational age, age-sex-specific birthweight centile and 5-minute Apgar score. Socioeconomic deprivation was assessed using the Welsh Index of Multiple Deprivation (WIMD) for the postcode of residence at delivery and then categorised into general population quintiles. WIMD is a standardized and validated tool for socioeconomic deprivation measurements established by Welsh government which consists of eight domains including income; employment; health; education; access to services; housing; community safety and physical environment.

The Appearance, Pulse, Grimace, Activity and Respiration (Apgar) score is a standardised assessment method to assess neonatal quality of life at the 1st and 5th minutes after birth. It consists of five components: skin colour, heart rate, reflexes, muscle tone, and breathing effort. Apgar score of 0 to 3 is considered as low, 4 to 6 as moderately abnormal and 7 to 10 as normal condition^17^. Low Apgar can independently influence neurodevelopmental child outcomes and can act as a possible mediator in the causal pathway between prenatal exposure to antihypertensive medication and neurodevelopmental outcomes.

The categorization of birth weight centiles into intervals was derived from clinical guidelines from the Royal College of Obstetricians and Gynaecologists and the National Institute for Health and Care Excellence. The selected cut-offs were chosen to balance clinical relevance with statistical robustness: they provide granularity to distinguish between foetal development (e.g. growth restriction vs. small for gestational age), while retaining a wide and stable reference group (21st–80th centiles) for comparative analyses. Foetal growth restriction was defined as “Fetal size or abdominal circumference < 3rd centile or < 10th centile with Doppler abnormalities”^18^. Small for gestational age was defined as “Fetal size < 10th centile”,^18^ while large for gestational age was defined as “there is no standardised definition of large for gestational age; it is often considered to mean a baby weighing more than the 90th birthweight centile”^19^.

### Statistical analyses

The referent category for all analyses was children born to mothers with no record of HDP nor receipt of anti-hypertensive medication. The groups compared with this category were children born to: mothers with any evidence of HDP (record of HDP or receipt of anti-hypertensive medication); mothers on anti-hypertensive medication (with or without a record of HDP); mothers with treated HDP (record of HDP and receipt of anti-hypertensive medication); and mothers with untreated hypertension (record of HDP but no record of anti-hypertensive medication). Antihypertensive medication was further categorized as: beta blockers, angiotensin-converting enzyme inhibitors, calcium channel blockers, diuretics, methyldopa, and angiotensin receptor blockers.

The characteristics of children born to mothers with and without any evidence of HDP were summarised using frequencies and percentages and compared using χ^2^ tests of association or trend. Generalized estimating equations with a binomial distribution and logit link function were used to model SEN and its subtypes, adjusting for correlations between serial measurements on the same pupil across multiple school years. The Quasi-likelihood under the Independence model Criterion (QIC) statistic was used to compare different correlation structures, with the structure with the lowest QIC value indicating the optimal model^20^. For ADHD, Cox proportional hazard models were used, with children followed from birth until the first occurrence of ADHD, death, emigration, or the end of follow-up (December 31, 2021), and each child contributing one record to the analysis. Analyses of all outcomes were conducted in a stepwise approach, beginning with univariate models, followed by partial adjustment for sociodemographic confounders, followed by full adjustment for sociodemographic, maternal and pregnancy confounders. Finally, the fully adjusted model was re-run including potential mediators. For non-ordered categorical variables (ethnicity, mode of delivery, and maternal smoking status) missing data were retained and analysed as separate categories. All analyses were conducted using STATA 18.

### Approvals

Ethical approval was obtained from the SAIL Information Governance Review Panel (IGRP) (Project 1089). Due to retrospective nature of the study, informed consent was waived by the SAIL Information Governance Review Panel (IGRP). This research was conducted in accordance with the Declaration of Helsinki.

## Results

### Cohort characteristics

Between 2009 and 2016, 200,152 children were born in Wales to mothers who met the inclusion criteria of continuous registration with a SAIL-participating general practice. Of these, 21,128 were excluded: 1,312 were stillborn and 19,816 could not be linked to Welsh educational records; (primarily due to migration out of Wales, attendance at private schools, or home education). The final cohort comprised 179,024 children (Supplementary Table S4 online). Among these 3440 (1.9%) were born to a mother with either a diagnosis of HDP or who received antihypertensive medication during pregnancy: 629 (0.3%) had treated HDP, 950 (0.5%) had untreated HDP, and 1861 (1.0%) were in receipt of anti-hypertensive medication but without a documented HDP diagnosis. Regarding specific medication exposures, 1828 (1.0%) offspring were exposed prenatally to beta-blockers, 163 (0.1%) to diuretics, 272 (0.2%) to calcium channel blockers, 249 (0.1%) to angiotensin-converting enzyme inhibitors, 489 (0.3%) to methyldopa, and 41 to angiotensin II receptor blockers. Compared to mothers without HDP or antihypertensive exposure, those with a diagnosis of HDP or who received anti-hypertensive medication were older, more likely to live in deprived areas, less likely to smoke, and more likely to be parous and have a multiple pregnancy (Table 1). Their offspring were more frequently delivered by Caesarean section and had lower 5-minute Apgar scores (Table 1).

**Table 1:** Characteristics of the study population by presence or absence of maternal hypertension disorders of pregnancy*.

	No maternal HDP *N* = 175,584 *N* (%)	Maternal HDP *N* = 3440 *N* (%)	Overall *N* = 179,024 *N* (%)	*p* value
*Child’s gender*				0.914
Female	85,965 (49)	1681 (48.9)	87,646 (49)	
Male	89,619 (51)	1759 (51.1)	91,378 (51)	
*Child’s ethnicity*				0.001
White	164,884 (93.9)	3182 (92.5)	168,066 (93.9)	
Asian	3473 (2)	79 (2.3)	3552 (2)	
Black	774 (0.4)	29 (0.8)	803 (0.4)	
Mixed	5266 (3)	121 (3.5)	5387 (3)	
Other	1071 (0.6)	28 (0.8)	1099 (0.6)	
Missing	116 (0.1)	1 (0)	117 (0.1)	
*Maternal age (years)*				< 0.001
<25	48,765 (27.8)	537 (15.6)	49,302 (27.5)	
25–29	51,850 (29.5)	851 (24.7)	52,701 (29.4)	
30–35	46,623 (26.6)	1013 (29.4)	47,636 (26.6)	
≥35	28,327 (16.1)	1039 (30.2)	29,366 (16.4)	
Missing	19	0	19	
*Maternal smoking*				< 0.001
Never smoker	73,371 (41.8)	1570 (45.6)	74,941 (41.9)	
Ex Smoker	2860 (1.6)	82 (2.4)	2942 (1.6)	
Quit during pregnancy	6158 (3.5)	141 (4.1)	6299 (3.5)	
Current smoker	29,911 (17)	546 (15.9)	30,457 (17)	
Missing	63,284 (36)	1101 (32)	64,385 (36)	
*Gestational age (weeks)*				< 0.001
<28	422 (0.2)	24 (0.7)	446 (0.3)	
28–32	1902 (1.1)	126 (3.7)	2028 (1.1)	
33–36	9577 (5.5)	453 (13.2)	10,030 (5.6)	
37	10,231 (5.8)	364 (10.6)	10,595 (5.9)	
38	20,608 (11.8)	615 (18)	21,223 (11.9)	
39	39,541 (22.6)	773 (22.6)	40,314 (22.6)	
40	48,763 (27.9)	638 (18.6)	49,401 (27.7)	
41	36,123 (20.7)	347 (10.1)	36,470 (20.5)	
≥42	7706 (4.4)	86 (2.5)	7792 (4.4)	
Missing	711	14	725	
*Mode of delivery*				< 0.001
Spontaneous vaginal	74,938 (42.7)	1124 (32.7)	76,062 (42.5)	
Assisted Delivery	13,577 (7.7)	243 (7.1)	13,820 (7.7)	
Breech vaginal	488 (0.3)	9 (0.3)	497 (0.3)	
Elective C/S	13,915 (7.9)	458 (13.3)	14,373 (8)	
Emergency C/S	18,382 (10.5)	586 (17)	18,968 (10.6)	
Missing	54,284 (30.9)	1020 (29.7)	55,304(30.9)	
*Parity*				< 0.001
0	73,755 (47.6)	1298 (42.9)	75,053 (47.5)	
1	50,078 (32.3)	935 (30.9)	51,013 (32.3)	
≥2	31,152 (20.1)	792 (26.2)	31,944 (20.2)	
Missing	20,599	415	21,014	
*5-minute Apgar score*				< 0.001
1–3	304 (0.2)	14 (0.4)	318 (0.2)	
4–6	1662 (1)	56 (1.7)	1718 (1)	
7–10	165,331 (98.8)	3184 (97.8)	168,515 (98.8)	
Missing	8287	186	8473	
*WIMD quintile*				0.034
1 (most Deprived)	46,386 (26.4)	956 (27.8)	47,342 (26.4)	
2	39,037 (22.2)	758 (22)	39,795 (22.2)	
3	34,596 (19.7)	664 (19.3)	35,260 (19.7)	
4	27,487 (15.7)	577 (16.8)	28,064 (15.7)	
5 (Least Deprived)	28,029 (16)	485 (14.1)	28,514 (15.9)	
Missing	49	0	49	
*Number of births*				< 0.001
1 (Singleton)	170,578 (97.1)	3271 (95.1)	173,849 (97.1)	
≥2 (Multiple)	5006 (2.9)	169 (4.9)	5175 (2.9)	
*Birth weight (centiles)*				< 0.001
1–3	5673 (3.2)	160 (4.7)	5833 (3.3)	
4–10	12,827 (7.3)	322 (9.4)	13,149 (7.4)	
11–20	17,782 (10.2)	397 (11.6)	18,179 (10.2)	
21–80	104,407 (59.8)	1888 (55.2)	106,295 (59.7)	
81–90	16,972 (9.7)	310 (9.1)	17,282 (9.7)	
91–97	11,855 (6.8)	224 (6.6)	12,079 (6.8)	
98–100	5157 (3)	117 (3.4)	5274 (3)	
Missing	911	22	933	

*Record of HDP or anti-hypertensive medication during pregnancy.

HDP hypertension disorders of pregnancy; N number; C/S Caesarean section; WIMD Welsh Index of Multiple Deprivation.

### Outcomes associated with HDP

Overall, 50,955 (28.5%) children had a record of SEN: 28,088 (15.7%) due to learning difficulties, 20,291 (11.3%) due to communication problems, 12,115 (6.8%) due to emotional difficulties, 3668 (2.1%) due to ASD, 3344 (1.9%) due to physical and medical difficulties, and 1977 (1.1%) due to sensory impairment. In addition, 3287 (1.8%) children were identified as having ADHD. The offspring of mothers with either a diagnosis of HDP or who received antihypertensive medication were more likely to have SEN overall (adjusted OR 1.47, 95% CI 1.35–1.59, *p* < 0.001), as well as SEN due to ASD (adjusted OR 1.49, 95% CI 1.16–1.92, *p* = 0.001), learning difficulties (adjusted OR 1.52, 95% CI 1.37–1.68, *p* < 0.001), communication problems (adjusted OR 1.44, 95% CI 1.28–1.62, *p* < 0.001), and physical or medical difficulties (adjusted OR 1.66, 95% CI 1.29–2.14, *p* < 0.001) (Table 2). These associations persisted after adjustment for sociodemographic, maternal, and pregnancy confounders. When further adjusted for potential mediators (birthweight, gestational age, and Apgar score), the associations were attenuated but remained significant for overall SEN (adjusted OR 1.32, 95% CI 1.21–1.44, *p* < 0.001), ASD (adjusted OR 1.46, 95% CI 1.12–1.89, *p* = 0.003), learning difficulties (adjusted OR 1.36, 95% CI 1.22–1.51, *p* < 0.001), and communication problems (adjusted OR 1.36, 95% CI 1.20–1.53, *p* < 0.001). Only the association with physical and medical difficulties became non-significant (adjusted OR 1.16, 95% CI 0.89–1.53, *p* = 0.260). No significant association was observed with ADHD (adjusted HR 0.97, 95% CI 0.71–1.31, *p* = 0.851).

**Table 2:** Associations between hypertension disorders of pregnancy* and childhood neurodevelopmental outcomes.

	Unadjusted *N* = 179,024			Adjusted for sociodemographic confounders ^1^ *N* = 178,975			Adjusted for sociodemographic, maternal and pregnancy confounders ^2^ *N* = 157,959			Adjusted for confounders and mediators ^3^ *N* = 151,262		
	OR	95% CI	P-value	OR	95% CI	P-value	OR	95% CI	P-value	OR	95% CI	P-value
Special educational need	1.33	1.23–1.43	< 0.001	1.34	1.24–1.44	< 0.001	1.47	1.35–1.59	< 0.001	1.32	1.21–1.44	< 0.001
ASD	1.53	1.21–1.93	< 0.001	1.53	1.21–1.93	< 0.001	1.49	1.16–1.92	0.001	1.46	1.12–1.89	0.003
Sensory impairment	1.34	0.98–1.84	0.065	1.34	0.98–1.85	0.062	1.36	0.97–1.92	0.071	1.17	0.81–1.67	0.386
Communication difficulties	1.37	1.23–1.53	< 0.001	1.37	1.23–1.53	< 0.001	1.44	1.28–1.62	< 0.001	1.36	1.20–1.53	< 0.001
Learning difficulties	1.38	1.26–1.51	< 0.001	1.39	1.26–1.53	< 0.001	1.52	1.37–1.68	< 0.001	1.36	1.22–1.51	< 0.001
Physical and medical difficulties	1.70	1.34–2.15	< 0.001	1.69	1.34–2.14	< 0.001	1.66	1.29–2.14	< 0.001	1.16	0.89–1.53	0.260
Emotional difficulties	1.04	0.89–1.21	0.592	1.03	0.88–1.21	0.637	1.15	0.97–1.37	0.085	1.14	0.96–1.35	0.128

	Unadjusted *N* = 160,261			Adjusted for sociodemographic confounders *N* = 160,234			Adjusted for sociodemographic, maternal and pregnancy confounders *N* = 141,940			Adjusted for confounders and mediators *N* = 135,933		
ADHD	0.95	0.72–1.24	0.732	0.95	0.73–1.25	0.742	1.05	0.78–1.41	0.723	0.97	0.71–1.31	0.851

*Record of HDP or anti-hypertensive medication during pregnancy.

OR odds ratio; CI confidence interval; N number; ASD autistic spectrum disorder; HR hazard ratio; ADHD attention deficit hyperactivity disorder.

^1^ Adjusted for child’s sex, ethnicity, age, and area deprivation; ^2^ Also adjusted for maternal age, maternal smoking, parity and multiple births; ^3^ Also adjusted for mode of delivery, gestational age, age-sex-specific birthweight centile and 5-minute Apgar score.

### Outcomes associated with untreated HDP, treated HDP and specific medications

**Fig. 1:**
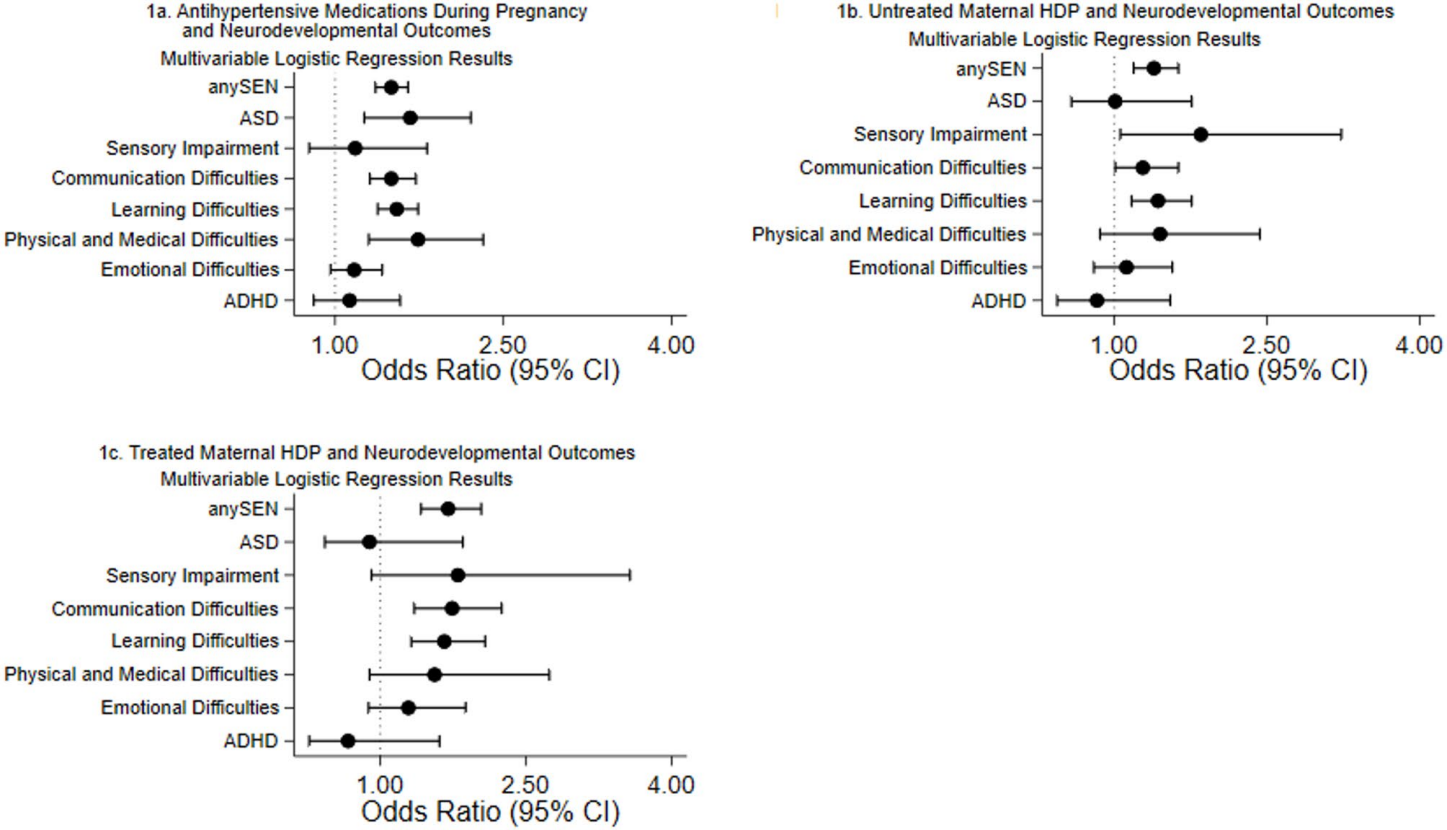
The association between maternal HDP or antihypertensive medications during pregnancy and neurodevelopmental outcomes. Forest plots showed adjusted odds ratios (OR) and 95% confidence intervals (CI) for the association between maternal HDP or antihypertensive medications during pregnancy and neurodevelopmental outcomes. Adjusted for child’s sex, ethnicity, age, area deprivation, maternal age, maternal smoking status, parity, and multiple births. The horizontal line represents 95% CIs and each point represent the adjusted ORs. The vertical line represents OR = 1, which indicates no association. (**a**) Description of subfigure 1a antihypertensive medications during pregnancy and neurodevelopmental outcomes. (**b**) Description of subfigure 1b untreated maternal HDP and neurodevelopmental outcomes. (**c**) Description of subfigure 1c treated maternal HDP and neurodevelopmental outcomes.

**Fig. 2:**
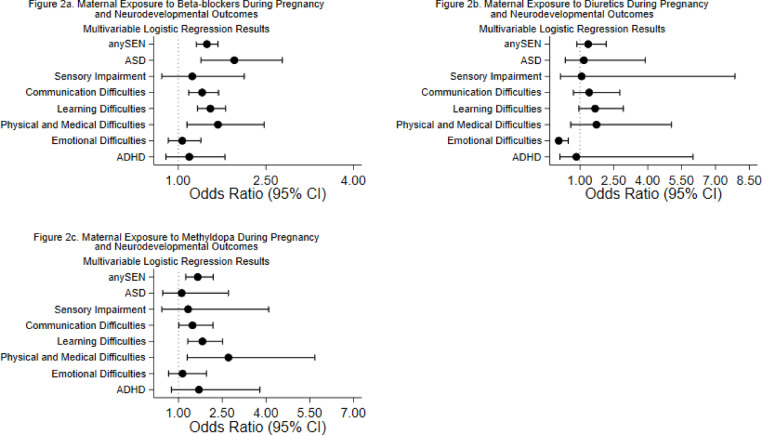
The association between maternal exposure to different classes of antihypertensive medications during pregnancy and neurodevelopmental outcomes. Forest plots showed adjusted odds ratios (OR) and 95% confidence intervals (CI) for the association between maternal exposure to different classes of antihypertensive medications during pregnancy and neurodevelopmental outcomes. Adjusted for child’s sex, ethnicity, age, area deprivation, maternal age, maternal smoking status, parity, and multiple births. The horizontal line represents 95% CIs and each points represent the adjusted ORs. The vertical line represents OR = 1, which indicates no association. (**a**) Description of subfigure 2a maternal exposure to beta-blockers during pregnancy and neurodevelopmental outcomes. (**b**) Description of subfigure 2b. maternal exposure to diuretics during pregnancy and neurodevelopmental outcomes. (**c**) description of subfigure 2c. maternal exposure to methyldopa during pregnancy and neurodevelopmental outcomes.

**Fig. 3:**
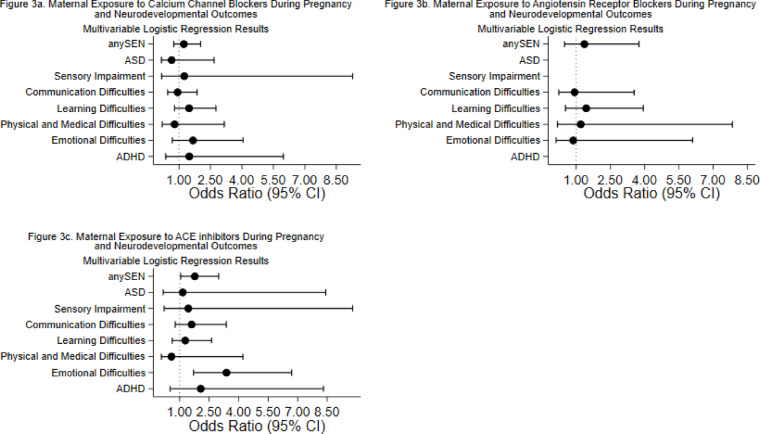
The association between maternal exposure to different classes of antihypertensive medications during pregnancy and neurodevelopmental outcomes. Forest plots showed adjusted odds ratios (OR) and 95% confidence intervals (CI) for the association between maternal exposure to different classes of antihypertensive medications during pregnancy and neurodevelopmental outcomes. Adjusted for child’s sex, ethnicity, age, area deprivation, maternal age, maternal smoking status, parity, and multiple births. The horizontal line represents 95% CIs and each points represent the adjusted ORs. The vertical line represents OR = 1, which indicates no association. (**a**) Description of subfigure 3a. maternal exposure to calcium channel blockers during pregnancy and neurodevelopmental outcomes. (**b**) Description of subfigure 3b. maternal exposure to angiotensin receptor blockers during pregnancy and neurodevelopmental outcomes. (**c**) Description of subfigure 3c. maternal exposure to ACE inhibitors during pregnancy and neurodevelopmental outcomes.

All-cause SEN and SEN attributed to learning difficulties and communication difficulties showed significant associations with prenatal exposure to antihypertensive medication (adjusted OR 1.50, 95% CI 1.36–1.65, *p* < 0.001; adjusted OR 1.55, 95% CI 1.38–1.74, *p* < 0.001; and adjusted OR 1.50, 95% CI 1.31–1.72, *p* < 0.001, respectively) (Fig. 1a) (Supplementary Table S5 online). These findings were consistent for the treated maternal hypertension group as well (Fig. 1c) (Supplementary Table S5 online). These outcomes were associated with prenatal exposure to both beta-blockers (adjusted OR 1.49, 95% CI 1.31–1.68, *p* < 0.001 for all-cause SEN) and methyldopa (adjusted OR 1.66, 95% CI 1.25–2.19, *p* < 0.001 for all-cause SEN) (Fig. 2a, c) (Supplementary Table S6 online) but importantly, were also significantly associated with untreated maternal HDP (adjusted OR 1.39, 95% CI 1.19–1.63, *p* < 0.001 for all-cause SEN) (Fig. 1b) (Supplementary Table S5 online). In contrast, SEN attributed to ASD showed a more specific pattern: it was significantly associated with prenatal exposure to antihypertensive medication (adjusted OR 1.67, 95% CI 1.26–2.21, *p* < 0.001) but not with untreated maternal HDP (adjusted OR 1.01, 95% CI 0.58–1.76, *p* = 0.960) (Fig. 1a, b) (Supplementary Table S5 online). Furthermore, this association with antihypertensive medication was specifically linked to beta-blocker exposure (adjusted OR 1.96, 95% CI 1.39–2.78, *p* < 0.001) (Fig. 2a) (see Supplementary Table S6 online), with no significant associations observed for other medication classes (Figs. 2b, c, 3a–c) (Supplementary Table S6 online).

## Discussion

In this large, unselected population cohort, ASD was associated with prenatal exposure to anti-hypertensive medication; specifically, beta-blockers. The association was independent of lifestyle, maternal and pregnancy confounders and appeared to be partially mediated by birth outcomes including birthweight, gestational age at delivery and Apgar score. Socioeconomic, maternal and pregnancy factors may influence maternal hypertension, adherence to treatment, and offspring neurodevelopmental outcomes, which may confound any treatment effects. For example, the incidence of pre-eclampsia and eclampsia are influenced by socioeconomic and cultural factors, access to perinatal and neonatal care is considerably limited in low- and middle-income countries (LMIC) compared to high-income countries,^21^ and only 8% of hypertensive mothers in LMIC have been reported to have a controlled blood pressure^22^.

A Prospective Urban Rural Epidemiological (PURE) conducted on 626 communities reported that only 13% of lower income communities have access to four antihypertensive classes compared to 94% of high-income communities^23^. Consistent with this, we observed that more deprived women were more likely to be diagnosed with HDP or receipt of anti-hypertensive medication. This suggests that economic constraints adversely affect treatment adherence and condition management. The most common antihypertensive medications used during pregnancy are methyldopa, labetalol, and nifedipine with different safety profiles^24^.

Importantly, ASD was not associated with untreated HDP, suggesting a potential medication-specific effect rather than an effect of the underlying condition. Our findings align with previous research from a U.S. case-control study that reported an association between treated maternal hypertension and ASD^12^. However, unlike that study, we did not observe an association between untreated hypertension and ASD.

In our study, other types of SEN, such as learning and communication difficulties, showed less specific associations with both methyldopa and beta-blocker exposure as well as untreated hypertension. This pattern suggests possible confounding by indication, where the medications may function as proxy measures for underlying HDP rather than exerting independent effects. A systematic review encompassing eight studies that included nearly 6,000 children supported this interpretation, concluding that neither methyldopa nor labetalol was associated with impaired neurocognitive development^14^. Similarly, a non-randomised trial examining methyldopa use in 195 pregnant women found no differences in sensory function, cognition, behaviour or IQ among exposed offspring at 4- and 7-years follow-up compared to unexposed children^25,26^. In a much smaller Canadian cohort study of 1,554 pregnancies, the association between HDP and cognitive delays was not statistically significant^27^. Therefore, our findings that both treated HDP and untreated HDP were associated with learning and communication difficulties contribute to the limited existing evidence.

We found no evidence that either HDP or anti-hypertensive medication were associated with ADHD in offspring. Our findings contrast with the limited existing evidence, primarily derived from small studies with methodological limitations. For instance, a Dutch study reported that ADHD was more common among 58 offspring whose mothers were treated with labetalol than among 83 whose mothers were treated with bed rest (OR 4.1, 95% CI 1.2–13.9)^11^. Similarly, another small cohort study, suggested an association between prenatal clonidine exposure (*n* = 22) and parental reports of hyperactivity and sleep disturbance, though no differences were observed in objective neurological findings or school performance metrics^28^.

Our study could not directly investigate the biological mechanisms through which beta-blockers might predispose to ASD. However, the association was attenuated after adjustment for birthweight, gestational age, and Apgar score, suggesting these factors may partially mediate the relationship. This aligns with established evidence that beta-blockers cross the placenta and can reduce placental blood flow, potentially affecting fetal oxygenation and nutrient transfer^27^. Previous research has linked beta-blocker exposure to intrauterine growth restriction and neonatal hypoglycemia^29^. These conditions, particularly when severe or prolonged, can adversely impact normal brain structure and neurodevelopment^27,29^.

Furthermore, beyond hypertensive disorders, several maternal and pregnancy factors demonstrate significant independent associations with childhood neurodevelopmental outcomes. Extensive research has linked smoking exposure to alterations in hypertension regulation, contributing to cardiovascular health disorders^30^. A multicentre Canadian cohort study revealed that maternal smoking was associated with poor neurodevelopmental outcomes among preterm offsprings, particularly affecting cognitive and motor development^31^. The risk persisted even after the adjustment for birth weight, gestational age, and other maternal factors.

Furthermore, a population-based study of 215,598 singleton pregnancies, examined the effect of hypertension status in conjunction with smoking habits. The study reported that maternal hypertension and smoking did not demonstrate a significant association with fetal growth^32^. Maternal smoking increased the risk of small for gestational age among all categories of hypertension conditions in term births compared to non- smokers. Another study reported that maternal hypertension was associated with bronchopulmonary dysplasia regardless of smoking status^33^. In our study, whilst those with HDP or who received antihypertensive medication were less likely to smoke, a significant association was found between HDP and neurodevelopmental outcomes suggesting both factors had independent pathways in affecting offspring development.

Apgar score is a potential early indicator for neonatal wellbeing and has been associated with several neurological and psychiatric disorders. In this study, women with HDP or who received antihypertensive medications had offsprings with lower Apgar score when compared to the comparison group. Modabbernia et al.^34^ found low and immediate Apgar score to be significantly associated with long-term ASD. However, the absolute risk remained small since the incidence rate of ASD was relatively low in the general population and the results were substantially confounded by birthweight and gestational age. Additionally, Hessen et al.^35^ conducted a longitudinal Australian study and reported that offsprings scoring 0–6 had 5.7 times greater odds of gross motor delays, while those scoring 7–8 had 4 times greater odds compared to offsprings with Apgar score of 10. Thus, Apgar score reinforces its value of being a crucial screening tool for early risk stratifications.

Our study found that those with a diagnosis of HDP or who received anti-hypertensive medication were older, consistent with multiple published studies^36–38^. One study observed that advanced maternal age was significantly associated with small for gestational age, miscarriage and pre-eclampsia^36^. Evidence suggests that women of advancing age are more likely to have higher risk of hypertension disorders during pregnancy whilst a meta-analysis demonstrated that women aged 35 or older had higher relative risk of having a child with autism compared to women aged 25–29 years^37^. Whilst advanced maternal age may increase the risk of HDP, the biological plausibility of this association is unclear^38^. It has been reported that a contributing factor to the incidence of pre-eclampsia is maternal cardiovascular adaptation dysfunction which could be due to complications of aging. This might compromise the placental function leading to adverse neurodevelopmental outcomes in the offspring. However, a large retrospective Canadian study of 2652 infants, demonstrated that advanced maternal age was not independently associated with neurodevelopmental outcomes among preterm births^39^. Thus, maternal age may not independently affect neurodevelopmental child outcomes.

Previous literature revealed that twin, triplets, or higher-order births were also independently associated with increased risk of neurodevelopmental outcomes. Multiple births can contribute to several biological mechanisms that increase risks, including prematurity, placental insufficiency, and perinatal complications. A cohort study of extremely preterm births (8,296 singleton, 2,164 twins, and 521 triplets) found that plurality was significantly associated with increased risk of death or neurodevelopmental outcomes compared to singleton births (OR 1.7, 95% CI 1.29–2.24). Thus, the current study supports these findings as singleton birth has a protective effect on neurodevelopmental outcomes compared to multiple births^40^.

Our unselected, population cohort provided a sufficiently large sample size to distinguish between effects of the underlying HDP condition and those attributable to antihypertensive medications, and to investigate specific medication classes. We investigated each drug class separately and ensured that no other antihypertensive medications were taken to avoid polytherapy/combination effects. We were able to analyse a range of neurodevelopmental outcomes, adjust for sociodemographic, maternal, and pregnancy-related factors, and explore potential mediators. The use of routinely collected healthcare and education data minimized selection bias and eliminated concerns regarding recall or reporting bias that often affect studies relying on retrospective self-reporting. Furthermore, a sensitivity analysis was conducted for the sub-group of women receiving antihypertensive medication who had no record of hypertension during the study window and the results were very similar to those observed for all women on antihypertensive medication.

Several limitations warrant consideration. Although we had comprehensive prescribing data, medication adherence cannot be confirmed, potentially introducing exposure misclassification. Beta-blockers and other antihypertensive agents are prescribed for multiple indications beyond hypertension, however our data lacked information on these alternative diagnoses. Also, we could not account for receipt of other medications and potential interactions in cases of polypharmacy. While the SAIL databank covers a substantial majority (83%) of Welsh family practitioners, complete population coverage was not achieved. Nevertheless, systematic differences between participating and non-participating practices in terms of the prevalence of HDP or SEN is unlikely, minimizing selection bias concerns. Despite adjusting for numerous sociodemographic, maternal, and pregnancy-related confounders, the possibility of residual confounding inherent to observational research remains, particularly regarding unmeasured factors such as severity of hypertension, maternal diet, or environmental exposures. Further future research needs to consider the subtypes of hypertension disorders during pregnancy- pre-existing hypertension and gestational hypertension as well as pre-eclampsia to examine how each type differently influences neurodevelopmental outcomes. Furthermore, timing (by trimester) or length of exposure of medications or dose of medication were not investigated. This is a limitation and should be a focus of future work.

Our findings revealed a significant association between prenatal exposure to beta-blockers and ASD. As beta-blockers remain the first-line therapy for HDP, and untreated HDP poses substantial maternal and fetal risks, these results have important clinical implications but should also be treated with caution. Further research should focus on replicating these findings, investigating biological mechanisms, evaluating alternative antihypertensive agents during pregnancy, and undertaking more causally focussed analyses. These data should inform physician-patient discussions regarding antihypertensive medication selection during pregnancy.

## Supplementary Information

Below is the link to the electronic supplementary material.

Supplementary Material 1

Supplementary Material 2

Supplementary Material 3

Supplementary Material 4

Supplementary Material 5

Supplementary Material 6

Supplementary Material 7

Supplementary Material 8

Supplementary Material 9

Supplementary Material 10

## Data Availability

The datasets generated and analysed during the study are not publicly available since the authors applied for permission to access, link, and analyse these data within the SAIL databank which is a secure safe haven environment. To do this, the researchers were required to undertake mandatory training in data protection, IT security and information governance. More information on these datasets is available from the corresponding author on reasonable request. Alternatively, interested researchers may apply directly to SAIL for data access to health and education data by emailing [SAILDatabank@swansea.ac.uk].
