# Correction: Association between prenatal exposure to antihypertensive medication and neurodevelopmental and educational outcomes in children

**DOI:** 10.1038/s41598-026-37334-z

**Published:** 2026-02-02

**Authors:** Shrifah Alkhalaf, Sarjit Singh, Jill P. Pell, Scott M. Nelson, Daniel F. Mackay, Michael Fleming

**Affiliations:** 1 School of Health and Wellbeing, University of Glasgow, Glasgow, UK; 2 School of Medicine, Dentistry and Nursing, University of Glasgow, Glasgow, UK; 3 School of Health and Wellbeing, College of Medical, Veterinary and Life Sciences, University of Glasgow, Clarice Pears Building, 90 Byres Road, Glasgow, G12 8TB UK

Correction to: *Scientific Reports* 10.1038/s41598-025-22887-2, published online 06 November 2025

The original version of this Article contained errors. Specifically in the introductory paragraphs and the Discussions section References were erroneously placed, cited or omitted. Additionally, in the second paragraph after the Abstract, the Articled erroneously quoted “50,000 fetal and neonatal deaths”, when the actual number is “500,000”. As a result:

The second paragraph after the Abstract,

“Chronic hypertension is a major risk factor for pre-eclampsia^6^ and, if untreated, can lead to life-threatening complications such as eclampsia, Hemolysis, Elevated Liver Enzymes, Low Platelet Count (HELLP) syndrome, and placental abruption^7^. Globally, pre-eclampsia contributes to over 50,000 fetal and neonatal deaths and 70,000 maternal deaths annually^8^, highlights the necessity of antihypertensive treatment during pregnancy. However, emerging evidence suggests that in-utero exposure to antihypertensive medication itself may influence neurodevelopmental trajectories in offspring.”

now reads:

“Chronic hypertension is a major risk factor for pre-eclampsia and, if untreated, can lead to life-threatening complications such as eclampsia, Hemolysis, Elevated Liver Enzymes, Low Platelet Count (HELLP) syndrome, and placental abruption^6,7^. Globally, pre-eclampsia contributes to over 500,000 fetal and neonatal deaths and 70,000 maternal deaths annually,^7^ highlighting the necessity of antihypertensive treatment during pregnancy. However, emerging evidence suggests that in-utero exposure to antihypertensive medication itself may influence neurodevelopmental trajectories in offspring^8^.”

Furthermore, the paragraph in Discussion section after Figure

“Our study could not directly investigate the biological mechanisms through which beta-blockers might predispose to ASD. However, the association was attenuated after adjustment for birthweight, gestational age, and Apgar score, suggesting these factors may partially mediate the relationship. This aligns with established evidence that beta-blockers cross the placenta and can reduce placental blood flow, potentially affecting fetal oxygenation and nutrient transfer^26^. Previous research has linked beta-blocker exposure to intrauterine growth restriction and neonatal hypoglycemia^6,29^. These conditions, particularly when severe or prolonged, can adversely impact normal brain structure and neurodevelopment^27,29^.

now reads:

“Our study could not directly investigate the biological mechanisms through which beta-blockers might predispose to ASD. However, the association was attenuated after adjustment for birthweight, gestational age, and Apgar score, suggesting these factors may partially mediate the relationship. This aligns with established evidence that beta-blockers cross the placenta and can reduce placental blood flow, potentially affecting fatal oxygenation and nutrient transfer^27^. Previous research has linked beta-blocker exposure to intrauterine growth restriction and neonatal hypoglycemia^29^. These conditions, particularly when severe or prolonged, can adversely impact normal brain structure and neurodevelopment^27,29^.”

The original Article has been corrected.

