# Supplementary material for: Association between prenatal exposure to antihypertensive medication and neurodevelopmental and educational outcomes in children
Source: Sci Rep. 2025 Nov 6;15:38929. doi: 10.1038/s41598-025-22887-2 (PMC12592423; doi:10.1038/s41598-025-22887-2)
Supplement: Supplementary file 1 — Supplementary Material 1 [file 41598_2025_22887_MOESM1_ESM.docx]

**A broader explanation for the data linkage**

The ADBE dataset contains information on all children born in Wales, however, it only includes unique child health ID numbers but not maternal health ID numbers. The NCCHD dataset contains information on all children in Wales, regardless of whether they were born in Wales or moved to Wales after they were born and includes both child and maternal ID numbers. These datasets were linked together using the unique child health ID numbers to enable variables to be used from both datasets, but more importantly to

1. Enable identification of children specifically born in Wales (those appearing on ADBE)

2. Enable further linkage of children in this birth cohort to corresponding maternal records (using the maternal health ID numbers available on NCCHD and held on other health datasets)

The birth records within the NCCHD/ADBE linked birth cohort were linked to:

- Maternal primary care records and prescriptions held within the WLGP dataset using the maternal health ID numbers present across all health databases

- Children’s primary care records and prescriptions held within the WLGP dataset using the child link numbers present across all health databases. ADHD diagnosis data was used from this dataset.

- Children’s school records held within the PLASC annual pupil census using the child link numbers.

The latter linkage of health data to children’s education records made use of a lookup file of unique health ID numbers to unique education ID numbers which was available in SAIL.

We did not use any secondary care data.

The WLGP dataset covers 83% of the Welsh population and contains attendance and clinical information for all general practice consultations including all patients’ demographics, investigations, diagnoses, prescribed medications, sign/symptoms and referrals. It includes national prescriptions in Wales from 2007).

WLGP datasets were linked to birth records within the NCCHD/ADBE which contains all maternal and child demographics, investigations, diagnoses, prescribed medications, sign/symptoms and referrals. The variables used from these linked datasets, child’s age, ethnicity, gender, maternal age, maternal smoking, parity, Apgar score, birthweight, mode of delivery, gestational age, and multiple birth.

EDUW datasets were linked to the above datasets and used to match on child health ID onto five different education files. The variables of interest on this linkage datasets (education ID, health ID, special educational needs (SEN) provision, age, and ethnicity). SEN provision means whether a child had any SEN or not. There were seven types of SEN analysed in addition to anySEN outcome, including ADHD, ASD, sensory impairments, communications difficulties, learning difficulties, physical and medical difficulties, and emotional difficulties. Also, deprivation data linked to the above datasets, which available within WIMD dataset. The Maternity Indicator Dataset (MIDS, containing additional obstetric and delivery information); used this data to replace missing data with MIDS data for some variables; maternal smoking, mode of delivery, parity, and Apgar score.

**Supplementary file S1: Broader explanation for the data linkage.**

The file includes information about each database and the methods used for linking the data across these databases.
