# Supplementary material for: Association between prenatal exposure to antihypertensive medication and neurodevelopmental and educational outcomes in children
Source: Sci Rep. 2025 Nov 6;15:38929. doi: 10.1038/s41598-025-22887-2 (PMC12592423; doi:10.1038/s41598-025-22887-2)
Supplement: Supplementary file 3 — Supplementary Material 3 [file 41598_2025_22887_MOESM3_ESM.docx]

**Supplementary Table S6**. Associations* between maternal anti-hypertensive medication class and childhood neurodevelopmental outcomes

|  | **Beta-blockers**  **N=156,294** | | **Diuretics**  **N=155,005** | | **Methyldopa**  **N=155,164** | | **Calcium-channel blockers**  **N=155,019** | | **Angiotensin receptor blockers**  **N=154,947** | | **ACE inhibitors**  **N=154,996** | |
| --- | --- | --- | --- | --- | --- | --- | --- | --- | --- | --- | --- | --- |
|  | OR (95% CI) | p-value | OR (95% CI) | p-value | OR (95% CI | p-value | OR (95% CI) | p-value | OR (95% CI) | P-value | OR (95% CI) | P-value |
| Special education needs | 1.49 (1.31-1.68) | <0.001 | 1.36 (0.86-2.16) | 0.177 | 1.66 (1.25-2.19) | <0.001 | 1.23 (0.75-2.03) | 0.404 | 1.36 (0.49-3.75) | 0.547 | 1.77 (1.05-2.98) | 0.030 |
| ASD | 1.96 (1.39-2.78) | <0.001 | 1.17 (0.35-3.89) | 0.786 | 1.11 (0.46-2.71) | 0.802 | 0.65 (0.16-2.67) | 0.560 | - | . | 1.16 (0.16-8.43) | 0.878 |
| Sensory  impairment | 1.24 (0.72-2.13) | 0.437 | 1.07 (0.14-7.86) | 0.940 | 1.33 (0.43-4.09) | 0.608 | 1.25 (0.17-9.28) | 0.821 | - | . | 1.44 (0.21-9.80) | 0.705 |
| Communication  difficulties | 1.41 (1.18-1.69) | <0.001 | 1.41 (0.71-2.76) | 0.317 | 1.48 (1.01-2.18) | 0.043 | 0.932 (0.46-1.86) | 0.842 | 0.93 (0.24-3.54) | 0.917 | 1.61 (0.77-3.37) | 0.198 |
| Learning  difficulties | 1.55 (1.33-1.81) | <0.001 | 1.67 (0.95-2.92) | 0.070 | 1.82 (1.32-2.51) | <0.001 | 1.48 (0.79-2.76) | 0.213 | 1.44 (0.53-3.94) | 0.470 | 1.28 (0.62-2.62) | 0.498 |
| Physical and  medical  difficulties | 1.68 (1.15-2.47) | 0.007 | 1.73 (0.59-5.05) | 0.310 | 2.71 (1.30-5.67) | 0.007 | 0.79 (0.20-3.16) | 0.746 | 1.20 (0.18-7.84) | 0.842 | 0.58 (0.07-4.22) | 0.591 |
| Emotional  difficulties | 1.07 (0.83-1.39) | 0.563 | 0.06 (0.01-0.48) | 0.006 | 1.14 (0.66-1.96) | 0.638 | 1.67 (0.68-4.06) | 0.256 | 0.87 (0.12-6.10) | 0.894 | 3.38 (1.70-6.69) | <0.001 |
|  | **N=140,435** | | **N=139,271** | | **N=139,411** | | **N=139,287** | | **N=139,221** | | **N=139,266** | |
| ADHD | 1.19 (0.79-1.80) | 0.391 | 0.84 (0.11-6.00) | 0.866 | 1.70 (0.76-3.79) | 0.194 | 1.49 (0.37-5.98) | 0.569 | - | . | 2.07 (0.51-8.31) | 0.301 |

*Adjusted for child’s sex, ethnicity, age, area deprivation, maternal age, maternal smoking status, parity, and multiple births

OR odds ratio; CI confidence interval; N number; ACE angiotensin converting enzyme; ASD autistic spectrum disorder; HR hazard ratio; ADHD attention deficit hyperactivity disorder.

**Supplementary Table S6: Associations between maternal anti-hypertensive medication class and childhood neurodevelopmental outcomes.**

This table shows adjusted odds ratios (OR) and 95% confidence intervals (CI) and p-values for the association between maternal exposure to different classes of antihypertensive medications during pregnancy and neurodevelopmental outcomes. Adjusted for child’s sex, ethnicity, age, area deprivation, maternal age, maternal smoking status, parity, and multiple births.
