# Supplementary material for: Association between prenatal exposure to antihypertensive medication and neurodevelopmental and educational outcomes in children
Source: Sci Rep. 2025 Nov 6;15:38929. doi: 10.1038/s41598-025-22887-2 (PMC12592423; doi:10.1038/s41598-025-22887-2)
Supplement: Supplementary file 6 — Supplementary Material 6 [file 41598_2025_22887_MOESM6_ESM.docx]

**Supplementary Table S5**. Associations* between treated and untreated hypertension disorders of pregnancy and childhood neurodevelopmental outcomes

|  | **Untreated HDP**  **N=155,762** | | | **Anti-hypertensive medication**  **N=157,131** | | | **Treated HDP**  **N=155,474** | | |
| --- | --- | --- | --- | --- | --- | --- | --- | --- | --- |
|  | OR | 95% CI | p-value | OR | 95% CI | p-value | OR | 95% CI | p-value |
| Special educational need | 1.39 | 1.19 – 1.63 | <0.001 | 1.50 | 1.36 – 1.65 | <0.001 | 1.70 | 1.42 – 2.04 | <0.001 |
| ASD | 1.01 | 0.58 – 1.76 | 0.960 | 1.67 | 1.26 – 2.21 | <0.001 | 0.89 | 0.43 – 1.85 | 0.767 |
| Sensory impairment | 1.85 | 1.06 – 3.23 | 0.029 | 1.18 | 0.77 – 1.82 | 0.423 | 1.80 | 0.91 – 3.57 | 0.090 |
| Communication difficulties | 1.28 | 1.01 – 1.63 | 0.039 | 1.50 | 1.31 – 1.72 | <0.001 | 1.74 | 1.35 – 2.25 | <0.001 |
| Learning difficulties | 1.43 | 1.17 – 1.76 | <0.001 | 1.55 | 1.38 – 1.74 | <0.001 | 1.66 | 1.32 – 2.08 | <0.001 |
| Physical and medical difficulties | 1.45 | 0.86 – 2.43 | 0.156 | 1.74 | 1.30 – 2.32 | <0.001 | 1.56 | 0.89 – 2.74 | 0.116 |
| Emotional difficulties | 1.12 | 0.80 – 1.57 | 0.487 | 1.172 | 0.96 – 1.42 | 0.107 | 1.29 | 0.88 – 1.88 | 0.176 |
|  | **Untreated HDP**  **N=139,951** | | | **Anti-hypertensive medication**  **N=141,197** | | | **Treated HDP**  **N=139,700** | | |
| ADHD | 0.83 | 0.44-1.55 | 0.569 | 1.13 | 0.81-1.58 | 0.446 | 0.67 | 0.27-1.61 | 0.374 |

*Adjusted for child’s sex, ethnicity, age, area deprivation, maternal age, maternal smoking status, parity, and multiple births

HDP hypertension disorders of pregnancy; N number; OR odds ratio; CI confidence interval; ASD autistic spectrum disorder; HR hazard ratio; ADHD attention deficit hyperactivity disorder.

**Supplementary Table S5: Associations between treated and untreated hypertension disorders of pregnancy and neurodevelopmental outcomes.**

This table shows adjusted odds ratios (OR) and 95% confidence intervals (CI) and p-values for the association between treated and untreated hypertension disorders of pregnancy and neurodevelopmental outcomes. Adjusted for child’s sex, ethnicity, age, area deprivation, maternal age, maternal smoking status, parity, and multiple births.
