# Supplementary material for: Association between prenatal exposure to antihypertensive medication and neurodevelopmental and educational outcomes in children
Source: Sci Rep. 2025 Nov 6;15:38929. doi: 10.1038/s41598-025-22887-2 (PMC12592423; doi:10.1038/s41598-025-22887-2)
Supplement: Supplementary file 7 — Supplementary Material 7 [file 41598_2025_22887_MOESM7_ESM.docx]

**Supplementary Table S4: Birth records in SAIL between 2009 to 2016 - Recodes of included and excluded in the study population**

| **Description** | **Number** |
| --- | --- |
| Number of births recorded between 2009 and 2016 | 336,629 |
| Number of births with no valid maternal link number (exclusion) | 23,513 |
| Number of births recorded between 2009 and 2016 | 313,116 |
| Number of live births not in Wales (exclusion) | 40,102 |
| Number of stillbirths (exclusion) | 1312 |
| Number of births recorded between 2009 and 2016 | 271,702 |
| Number of mothers with incomplete SAIL coverage (exclusion) | 72,862 |
| Number of births in Wales between 2009 and 2016 to mothers with full SAIL coverage | 198,840 |
| Number of children with no follow up education data | 11,861 |
| Number of births in Wales between 2009 and 2016 to mothers with full SAIL coverage and where the child has education data recorded | 186,979 |
| No. of children with invalid education records – child less than 4 years old or attending school out with study period (exclusion) | 7,955 |
| Number of births in Wales between 2009 and 2016 to mothers with full SAIL coverage and where the child has valid education data recorded | 179,024 |

**Supplementary Table S4: Birth records in SAIL between 2009 to 2016.**

This file shows the process of selecting the study population. It illustrates the initial identified records, the inclusion and exclusion criteria, and the final sample size.
