# Supplementary material for: Association between prenatal exposure to antihypertensive medication and neurodevelopmental and educational outcomes in children
Source: Sci Rep. 2025 Nov 6;15:38929. doi: 10.1038/s41598-025-22887-2 (PMC12592423; doi:10.1038/s41598-025-22887-2)
Supplement: Supplementary file 8 — Supplementary Material 8 [file 41598_2025_22887_MOESM8_ESM.docx]

**Supplementary Figure 2.** Visual summary of key findings

Treated

hypertension

Anti-hypertensive medication

Untreated hypertension

950

629

1,861


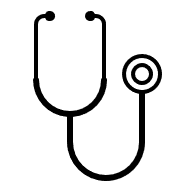

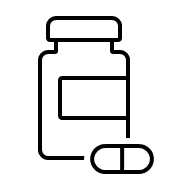


39%↑ Special educational needs

43%↑ Learning difficulties

50%↑ Special educational needs

50%↑ Learning difficulties

**67%↑ ASD (96%↑ for beta-blockers)**

Specific effect

Due to medication

Non-specific effect

Due to disease

**Figure legend:**

This figure displays the key findings of the study, showing the association between prenatal exposure to antihypertensive medication and neurodevelopmental and educational outcomes in children across all comparison groups. Percentage risks are presented based on the adjusted odds ratios.
