# Supplementary material for: Association between prenatal exposure to antihypertensive medication and neurodevelopmental and educational outcomes in children
Source: Sci Rep. 2025 Nov 6;15:38929. doi: 10.1038/s41598-025-22887-2 (PMC12592423; doi:10.1038/s41598-025-22887-2)

Supplementary Figure 1. Pictorial abstract.

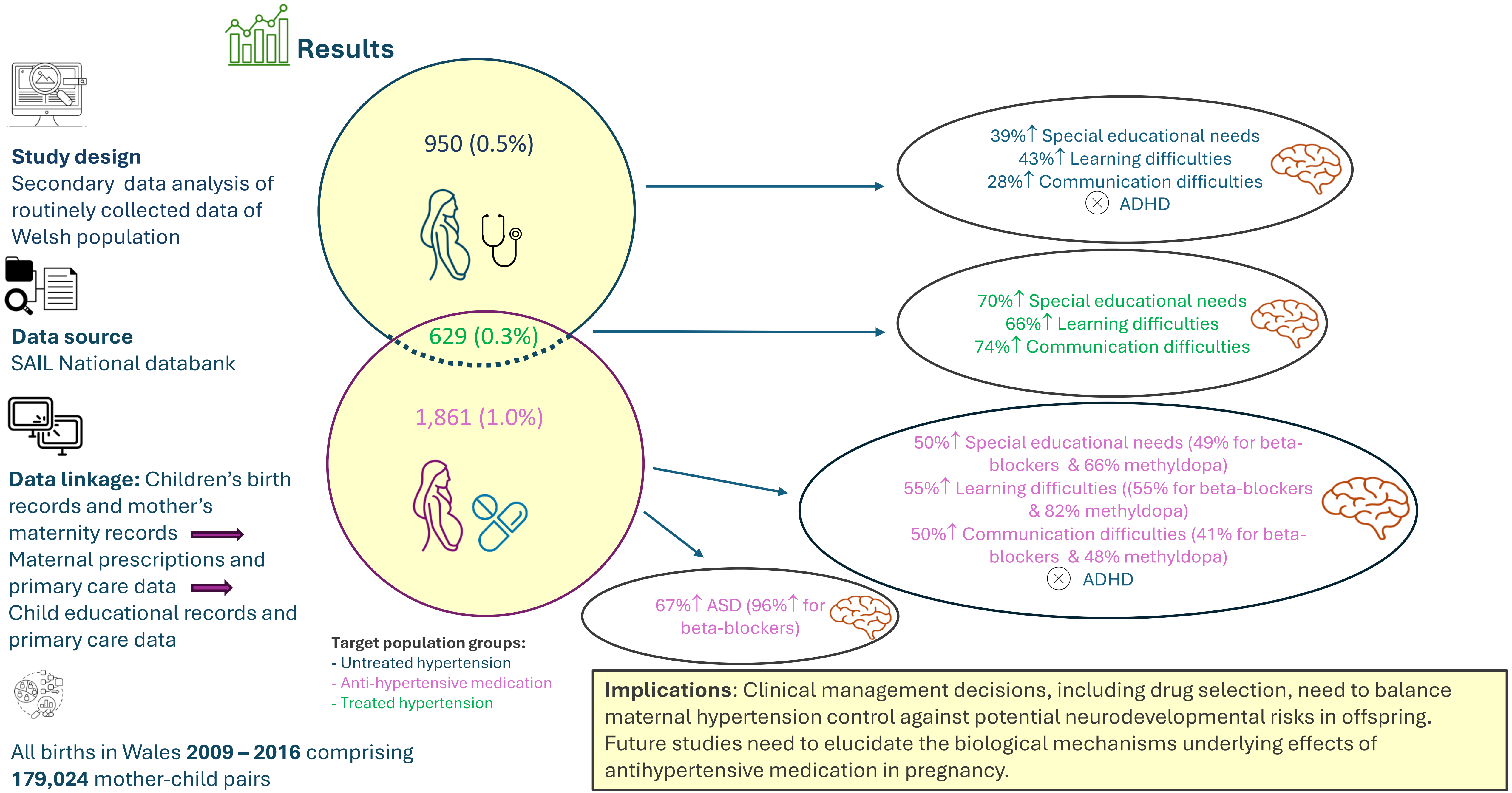

Supplement: Supplementary file 9 — Supplementary Material 9 [file 41598_2025_22887_MOESM9_ESM.pdf]
