# Supplementary material for: Association between prenatal exposure to antihypertensive medication and neurodevelopmental and educational outcomes in children
Source: Sci Rep. 2025 Nov 6;15:38929. doi: 10.1038/s41598-025-22887-2 (PMC12592423; doi:10.1038/s41598-025-22887-2)
Supplement: Supplementary file 10 — Supplementary Material 10 [file 41598_2025_22887_MOESM10_ESM.docx]

**Supplementary Figure 1.** Pictorial abstract.

Pictorial abstract illustrating the study design, main results, and clinical implications of a retrospective analysis examining the use of antihypertensive medication during pregnancy and its impact on longer-term neurodevelopmental outcomes in children.

**Supplementary Figure 2.** Visual summary of key findings

This figure displays the key findings of the study, showing the association between prenatal exposure to antihypertensive medication and neurodevelopmental and educational outcomes in children across all comparison groups. Percentage risks are presented based on the adjusted odds ratios.
